# Urban living influences the nesting success of Darwin’s finches in the Galápagos Islands

**DOI:** 10.1002/ece3.7360

**Published:** 2021-03-16

**Authors:** Johanna A. Harvey, Kiley Chernicky, Shelby R. Simons, Taylor B. Verrett, Jaime A. Chaves, Sarah A. Knutie

**Affiliations:** ^1^ Department of Ecology and Evolutionary Biology University of Connecticut Storrs CT USA; ^2^ Department of Biology San Francisco State University San Francisco CA USA; ^3^ Colegio de Ciencias Biológicas y Ambientales Universidad San Francisco de Quito Laboratorio de Biología Evolutiva Diego de Robles y Pampite Quito Ecuador; ^4^ Institute for Systems Genomics University of Connecticut Storrs CT USA; ^5^ Present address: Division of Invertebrate Zoology American Museum of Natural History New York NY USA

**Keywords:** anthropogenic debris, dry year, entanglement, Galápagos Islands, *Geospiza fuliginosa*, La Nińa, nest material, trash, urban ecology

## Abstract

Urbanization is expanding worldwide with major consequences for organisms. Anthropogenic factors can reduce the fitness of animals but may have benefits, such as consistent human food availability. Understanding anthropogenic trade‐offs is critical in environments with variable levels of natural food availability, such as the Galápagos Islands, an area of rapid urbanization. For example, during dry years, the reproductive success of bird species, such as Darwin's finches, is low because reduced precipitation impacts food availability. Urban areas provide supplemental human food to finches, which could improve their reproductive success during years with low natural food availability. However, urban finches might face trade‐offs, such as the incorporation of anthropogenic debris (e.g., string, plastic) into their nests, which may increase mortality. In our study, we determined the effect of urbanization on the nesting success of small ground finches (*Geospiza fuliginosa*; a species of Darwin's finch) during a dry year on San Cristóbal Island. We quantified nest building, egg laying and hatching, and fledging in an urban and nonurban area and characterized the anthropogenic debris in nests. We also documented mortalities including nest trash‐related deaths and whether anthropogenic materials directly led to entanglement‐ or ingestion‐related nest mortalities. Overall, urban finches built more nests, laid more eggs, and produced more fledglings than nonurban finches. However, every nest in the urban area contained anthropogenic material, which resulted in 18% nestling mortality while nonurban nests had no anthropogenic debris. Our study showed that urban living has trade‐offs: urban birds have overall higher nesting success during a dry year than nonurban birds, but urban birds can suffer mortality from anthropogenic‐related nest‐materials. These results suggest that despite potential costs, finches benefit overall from urban living and urbanization may buffer the effects of limited resource availability in the Galápagos Islands.

## Graphical abstract

Urbanization is expanding worldwide with major consequences for organisms, understanding the trade‐offs of urbanization is of critical importance, particularly in environments with unreliable annual natural food availability, such as the Galápagos Islands. Our study examined the effect of a dry year on the reproductive effort and success of Darwin's finches. We found that urban birds have overall higher reproductive success than nonurban birds, but urban birds can suffer a negative consequence by using anthropogenic‐related nest‐materials, suggesting that despite the potential cost of urban living, finches benefit overall from urban living and urbanization may buffer the effects of limited resource years. Photo credit: Kiyoko Gotanda

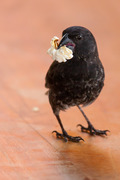

## 1 INTRODUCTION

Few places remain unaltered by humans with increasing urbanization now impacting nearly all ecosystems (Vitousek, 1997). Urbanization, the concentration of human populations resulting in altered landscapes, can directly change the physical structure of the ecosystem through the creation of roads and buildings and introducing artificial light, pollution, and noise (Dominoni et al., 2013; Fernández‐Juricic, 2002; Herrera‐Dueñas et al., 2017). Consequently, native fauna can suffer reduced fitness or extirpation in response to urbanization‐related stressors, such as environmental change, increased predation, limited natural food availability, and increased disease and parasites (Bailly et al., 2016; Blair, 1996; Johnson & Munshi‐South, 2017; Lepczyk et al., 2004). However, urban living can also benefit organisms by reducing the natural predation risk and increasing alternative resource availability, such as human food sources and habitat structures (Gering & Blair, 1999; Lowry et al., 2013; Møller et al., 2015). The effect of anthropogenic materials on seabirds has been well examined (Roman et al., 2019); however, the impacts on passerines, particularly on nesting success in urban areas, have not been well assessed. The effect of urbanization on birds can vary, but include earlier lay dates and lower reproductive success in urban versus nonurban areas (Chamberlain et al., 2009; Sepp et al., 2018). Urban food availability has been suggested as a principal factor driving the variation of demographic responses across passerines (Chamberlain et al., 2009). While species diversity can decline in urban areas (Kark et al., 2007), urban areas still sustain a number of native species (Aronson et al., 2014); this duality presents an opportunity to better understand the trade‐offs experienced by a species in response to urbanization.

Determining the effects of urbanization on islands is especially important given that islands host 20% of all terrestrial plant and vertebrate species diversity (Courchamp et al., 2014; Kier et al., 2009). Furthermore, island endemic species, particularly specialist species, can be highly sensitive to natural and anthropogenic perturbations (Buckley & Jetz, 2007) due to their small population sizes, low immigration, and associated genetic factors (Benning et al., 2002). One of the few existing studies on island endemics adapting to urbanization is on Caribbean reptiles which persist in urban environments but in lower numbers (Jesse et al., 2018). An example from a nonisland specialist species is the urban adapted dark‐eyed juncos (*Junco hyemalis*), for which longer breeding seasons in urban areas result in higher reproductive success (Yeh & Price, 2004). The limited number of existing island urbanization studies suggests that native species may differ in their responses to urbanization and shows that a clearer understanding of endemic island species response to urbanizations is needed. As island species face extinction threats on many fronts, examining trade‐offs for urban animals on islands could provide insight into their ability to respond to anthropogenic pressures or help inform management and conservation of the species.

The Galápagos Islands of Ecuador have experienced recent urbanization due, in large part, to growth in ecotourism. Since the 1990s, Galápagos tourism has increased by an average of 9.4% per year, with current estimates of nearly 225,000 visiting tourists each year. The resident human population has increased by an average of 6.4% per year since the early 1990’s, reaching 25,244 in 2015 (Epler, 2007; Walsh & Mena, 2016). The recent human population growth and associated urbanization of the Galápagos islands provides an ideal “laboratory” to determine the effects of human activity on endemic animals. For example, recent studies have shown that Darwin's finches in urban areas prefer nonnatural food compared to finches in nonurban areas (De León et al., 2018), resulting in changes to their microbiota (Knutie et al., 2019), epigenetics (McNew et al., 2017), and morphology (De Léon et al., 2011; Hendry et al., 2006).

The Galápagos also face natural stressors, such as highly variable climatic conditions. The islands have a hot, wetter season from approximately January to May, and a cool, drier season from approximately June to December (Grant & Boag, 1980). The conditions during these seasons depend on the Inter‐Tropical Convergence Zone (ITCZ) and the periodically irregular El Niño Southern Oscillation (ENSO) (Trueman & d’Ozouville, 2010). El Niño events can often result in wetter seasons with high primary productivity and therefore high food resources for the finches, whereas La Niña events are characterized by drier seasons with limited primary productivity and food resources (Grant & Boag, 1980; Trueman & d’Ozouville, 2010). Consequently, low reproductive fitness has been a consistent documented pattern reported in Darwin's finches across the Galápagos islands in dry La Niña years, with this effect being more pronounced in the arid coastal zones (Boag & Grant, 1981; Gibbs & Grant, 1987; Grant & Grant, 1989, 1999; Koop, LeBohec, & Clayton, 2013). Low reproductive success in response to dry years has also been found in other island land birds, such as Galápagos mockingbirds (Curry & Grant, 1989; McNew et al., 2019). To date, studies have not examined the influence of urbanization on the reproductive fitness of Darwin's finches during dry conditions. Therefore, the Galápagos islands present a unique opportunity to examine the effects of growing, yet incipient, urbanization in a landscape where climate could be further exacerbating the positive or negative effects of urbanization on an endemic species.

In our study, we examined the effect of urbanization on the reproductive effort and nesting success of small ground finches (*Geospiza fuliginosa*; a species of Darwin's finch) during a La Niña year. First, we determined whether reproductive effort (i.e., nests built, eggs laid, hatchlings) and success (i.e., young fledged) of small ground finches differed between urban and nonurban areas by tracking the survival of nests from construction to egg laying, hatching, nestling survival, and confirmed fledging of young. During years with dry conditions, the reproductive effort and success of Darwin's finches is lower than in years with wet conditions, which has been linked to reduced natural food availability (Boag & Grant, 1981; Koop, LeBohec and Clayton, 2013). Because urban areas are supplemented with additional human food resources (De León et al., 2018), finch reproductive effort and success is predicted to increase in urban areas compared to nonurban areas. However, finches incorporate human‐related debris into their nest (Knutie et al., 2014; Theodosopoulos & Gotanda, 2019). Debris can include plastic, fishing line, human hair, synthetic string, paper, etc. which are readily available in many urban areas and can result in injury (Jiguet et al., 2019) or even death due to entanglement (Jagiello et al., 2018; Theodosopoulos & Gotanda, 2019; Townsend & Barker, 2014). Therefore, although urban finches are predicted to have higher overall reproductive success, urban finches likely face a trade‐off related to anthropogenic debris in their nests: urban finches may benefit from urban resource availability but may also suffer negative consequences (i.e., entanglement, ingestion) due to anthropogenic debris use.

## 2 MATERIALS AND METHODS

### 2.1 Study system

We conducted our study between February and May 2018 (during the breeding season) in the arid lowland climatic zone of San Cristóbal (557 km^2^) in the Galápagos Islands. Breeding for ground finches is initiated by heavy rainfall events and continued breeding is dependent on continued rainfall (Boag & Grant, 1984; Kleindorfer, 2007). Rainfall on San Cristóbal is highly variable, with interannual variation alternating between high and low rainfall (Grant & Boag, 1980).

**FIGURE 1:**
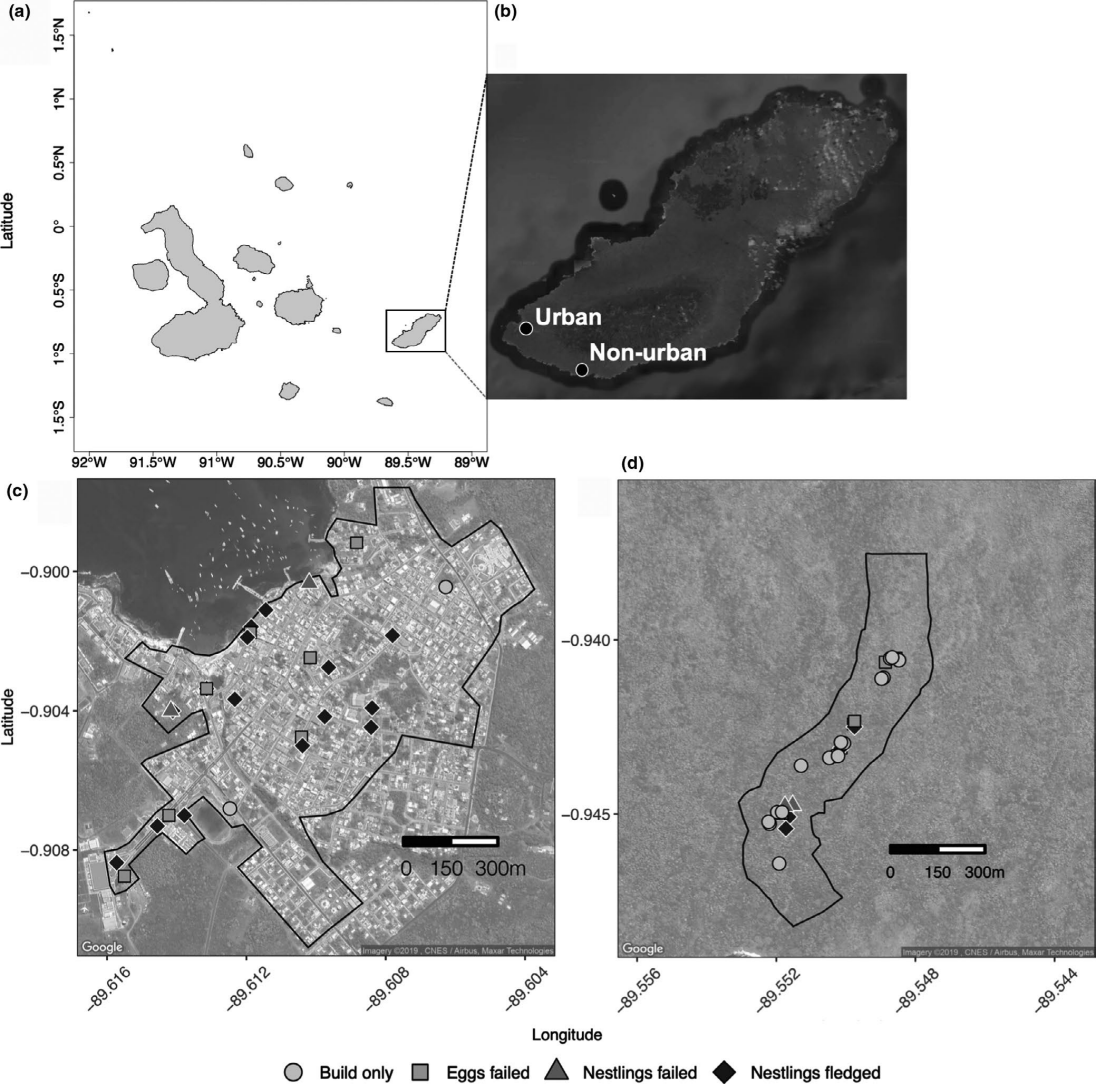
(a) Map of the major islands of the Galápagos archipelago and inset map of (b) San Cristóbal Island with sampling areas noted (black dots) for the urban area (Puerto Baquerizo Moreno) and the nonurban area (Jardín de Opuntias). Satellite maps of the (c) urban area and (d) the nonurban area showing nests which were builds only (gray circle), nests with eggs that failed (gray square), nests with nestlings that failed (dark gray triangle), and nests with nestlings that fledged (black diamond) across each sampling site with the search area delineated by the black polygon border. Map data Google Maps Imagery © 2019 and the GADM database (Hijmans et al., 2014)

We quantified nest building, egg laying, hatching, and fledging of small ground finches in an urban and nonurban area. The urban area was in the capital city of Puerto Baquerizo Moreno (hereon, urban area), which is the second largest city in the Galápagos archipelago with a human population of 6,553 (INEC, 2015). The urban area consists of an urban matrix which hosts a concentrated human population where land has been altered for human usage and consists of primarily impermeable concrete or stone surfaces, structures, and roads. Our urban study area measured 0.79 km^2^ (~1.2 km by 0.62 km) and included tourist and residential zones (Figure 1c). The search area within the urban study area was delineated by the urban matrix and excluded large undeveloped habitats on the outskirts of Puerto Baquerizo Moreno. The nonurban area was in the Jardín de Opuntias (hereon, nonurban area), which is a Galápagos National Park site located eight km southeast of the urban area consisting of vegetated natural habitats with no unnatural impermeable surfaces present. Our nonurban study area measured 0.21 km^2^ and covered 1.4 km of the main trail and 0.15 km to each side (Figure 1d). The search area is larger in the urban area than the nonurban area due to spatial mismatch and differences in environmental structure, which can result in urban patches devoid of suitable nesting areas. Search efforts, via total number of search hours per person for each day across sites, were tracked for each study area to normalize search efforts. Our nonurban area, the Jardín de Opuntias, is named for the large presence of the arboreal cactus, *Opuntia megasperma* which is one of the preferred nesting locations of small ground finches. However, cacti are rare across San Cristóbal, likely due to destruction by introduced mammals in the 1800s, but are locally abundant within the Jardín de Opuntias (Dvorak et al., 2019; Phillips et al., 2012). Small ground finch nests are commonly found in cacti as well as trees such as matazarno (*Piscidia cathagenensi*) and Galápagos acacia (*Acacia rorudiana*), and nests are common in both the nonurban and urban area. The nonurban area receives very low human visitation: locals occasionally, but rarely, visit the site to access the beach. Cacti are also frequently cultivated in urban areas and are found in garden beds, planters, city parks, and the main boardwalk. Urban finches nest in native and nonnative trees, human‐planted *Opuntia* cacti, and occasionally in human built structures, such as gutters and building signs.

### 2.2 Locating nest sites and data collection

In each urban and nonurban area, we searched intensively for nests and for small ground finches exhibiting nest‐building behaviors, including vocalization and behavioral cues. The field sites were searched nearly every other day for evidence of nest‐building activity by small ground finches. We followed all nest builds that were accessible with the use of a 10‐foot (~3 meter) ladder. Once found, nests were checked every other day with observations made primarily through binoculars to minimize nest disturbance and secondarily through a small camera (Contour LLC, Provo, USA) attached to an extendable pole when the nest was not attended by adults. Once the eggs hatched, we followed the survival of nestlings and banded them with a unique color band combination when they were 7–8 days of age (hatch date = day 0). Successful fledging was confirmed by resighting and identification of color‐banded nestlings two to seven days after nestlings have left the nest, as in previous studies (Knutie et al., 2016). After nestling birds fledged or died, the nest was collected and placed in a sealed plastic bag. Each nest was carefully dissected to separate natural and anthropogenic materials, after which each material type was weighed (g). Anthropogenic nest materials were then qualitatively identified (composition and possible source material) in order to quantify nest materials which are preferentially incorporated into nests and those that may be associated with trash‐related mortality. All detected nest failures and mortalities were documented, and causation was determined when possible. Materials associated with mortality via ingestion or entanglement were also identified and documented.

Our study resulted from a single year of sampling, and no building or breeding individuals in our study had been previously banded. We did not observe any dispersal of banded birds across urban and nonurban areas during the study period. The flight distance between urban and nonurban sampling areas is eight km, and previous studies have not found dispersal in small ground finches to occur across habitats (Kleindorfer et al., 2006). Therefore, it is unlikely that small ground finches forage across the study areas.

### 2.3 Statistical analyses

All data were analyzed in Rstudio v1.2 (R Core Team, 2012). We calculated daily total search hours by multiplying the number of hours searched by the number of people searching for each day at each site (urban and nonurban, respectively) across all days of the survey period. We tested survey effort, to determine if search effort was equivalent across sites, using an independent *t* test on daily total search hours at each site after examining data for homogeneity of variance using a Fligner‐Killeen test and Q‐Q plots for assessment of normality.

We used General Linear Models (McCullagh & Nelder, 1989) using the *glm* base R function and ANOVAs using the *car* package (Fox & Weisberg, 2011). We first verified that data met assumptions of models by checking for overdispersion and underdispersion. We used three different GLM models with a binomial error structure to determine whether location (urban and nonurban) affected the following predictor variables in terms of binary presence/absence of (*i*) eggs in detected nests, (*ii*) nests with nestlings, and (*iii*) presence of trash in nest material. For each analysis, responses are presence/absence (1’s and 0’s, respectively). Additionally, we used binomial logistic regressions, which are a special case of GLM, to determine the effect of location on (*iv*) hatching and (*v*) nestling survival trials independently. The response variable is a matrix of trials, which are successes and failures, where the number of eggs hatched and nestling fledged per nest represent successes while eggs not hatched and nestling mortalities represent failures, respectively. We also used a binomial logistic regression to determine the effect of location (urban and nonurban) along with the proportion of (*vi*) anthropogenic debris in the nest on nestling survival, measured as nestling successes and failures per nest.

## 3 RESULTS

Nest searching was conducted over 49 days in the urban area with an average of 12.51 ± 4.21 daily total search hours (sum of hours searched by each person on the search team). Nest searching was conducted over 40 days in the nonurban area with an average of 12.23 ± 7.5 daily total search hours. The number of search hours did not significantly vary across the urban and nonurban study areas (Independent *t* test, t* = *−0.22, *df* = 75.51, *p* = .82).

**FIGURE 2:**
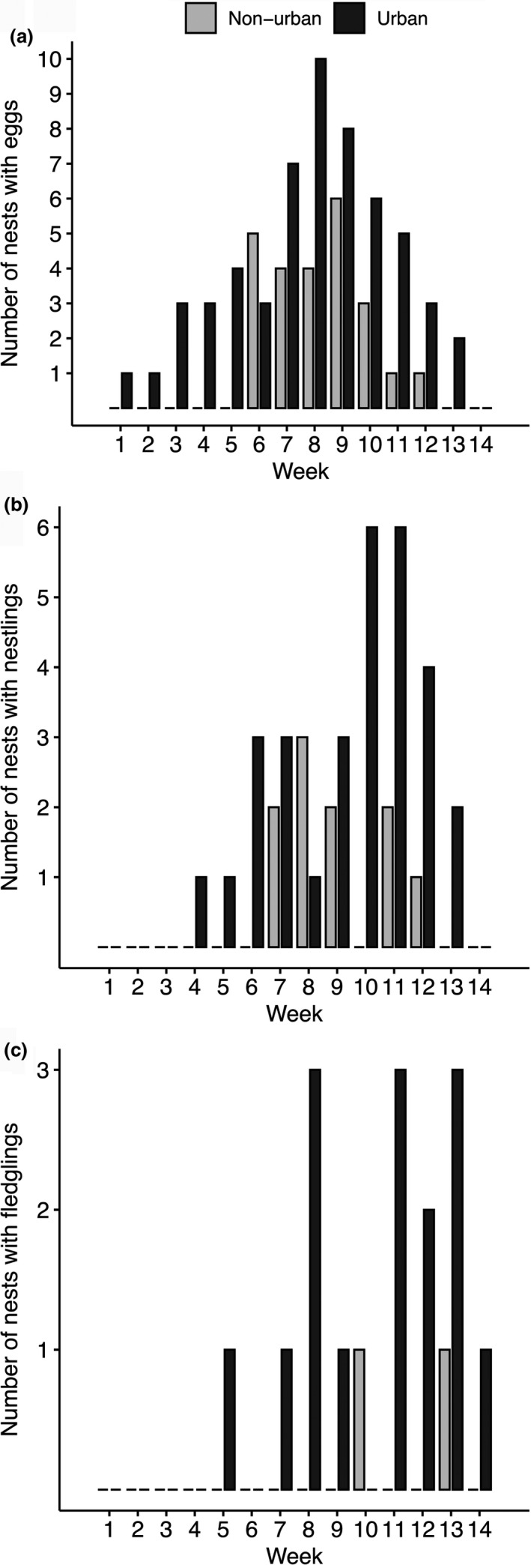
Histograms of reproductive activity noting frequency of nests with (a) eggs, (b) nestlings, and (c) fledgling (where fledging occurred that week) across each week of the study period, weeks 1–14, in nonurban (gray) and urban (black) sampling areas

The first urban finch nest with eggs was found on 10 February 2018, whereas the first nonurban finch nest with eggs was found on 7 March 2018 (Figure 2). The last urban nest with eggs was found on 14 April 2018 and the last nonurban finch nest with eggs was found on 13 April 2018, resulting in a breeding season of 68 days in the urban area and 37 days in the nonurban area. Small ground finches built 29 nests in the urban area and 29 nests in the nonurban area (Figure 1, Table 1). The urban area had more nests with eggs (*n* = 25 nests with eggs out of 29 nests built) than the nonurban area (*n* = 12 nests with eggs out of 29 nests built) (*i*, GLM, *χ^2^ = *13.28, *df = *1, *p* < .0001).

**TABLE 1:** Nesting effort of small ground finches in nonurban and urban areas

Variable	Nonurban	Urban
No. built	29	29
No. with eggs	12/29 (41.3%)	25/29 (86.2%)
No. with nestlings	6/12 (50.0%)	17/25(68%)
No. with at least one fledgling	2/6 (33.3%)	15/17 (88.2%)
No. with anthropogenic materials	0/10 (0%)	22/22 (100%)

The urban nests were more likely to contain hatchlings (*n* = 17 nests with hatchling from 29 nests built) than the nonurban nests (*n* = six nests with hatchlings from 29 nests built) (*ii*, GLM, *χ^2^ = *9.0, *df = *1, *p* = .003). Of the 25 urban nests with eggs, eight failed at the egg stage. Three nests were damaged during likely predation events, with no remains found and the nest entrance destroyed. Two nests were found abandoned with eggs intact and cold. One nest had only one egg and was found infested by ants. One nest was found with a single egg ejected and the remaining eggs found cold in the unattended nest. One nest was found empty with no evidence of predation or cause of failure determined. Of the 12 nonurban nests with eggs, six failed at the egg stage. One nest had clear signs of predation with a broken eggshell found outside of the nest. One nest was found empty with no evidence to explain its failure. The remaining four failed nests were abandoned, with cold eggs found in the unattended nest.

**FIGURE 3:**
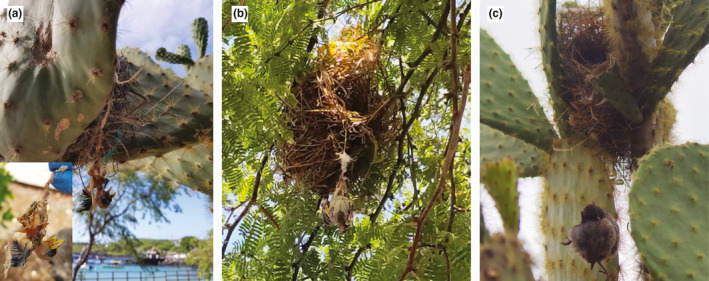
Documented small ground finch mortalities due to ingestion/entanglement of anthropogenic nest materials: (a) 6‐day old nestling ingestion/entanglement with plastic and human hair, (b) 12‐day old nestling entanglement with synthetic string, (c) adult female entanglement during nest building with human hair

Overall survival, from egg stage and fledging, was higher in the urban compared to the nonurban area: urban nests were more likely to have eggs that hatched (*iv*, *χ^2^ = *4.34, *df = *1, *p* = .04) and nestlings that fledged (*v*, *χ^2^ = *14.35, *df = *1, *p* = .0002) than the nonurban nests. Only two urban nest failures occurred during the nestling stage. One nest was found with a six‐day old dead nestling hanging from ingested hair that was woven into the nest (Figure 3a) and the two other nestlings missing from the nest. One nest was found empty with no apparent cause for mortality or nestling remains found. For three out of the four nest failures during the nestling stage in the nonurban area, nestlings were missing with no clear signs of depredation. These mortalities occurred at six‐, seven‐, and ten days posthatching. One nonurban nest had signs of depredation, with partial nestling remains (i.e., limbs and skull) found near the nest.

**FIGURE 4:**
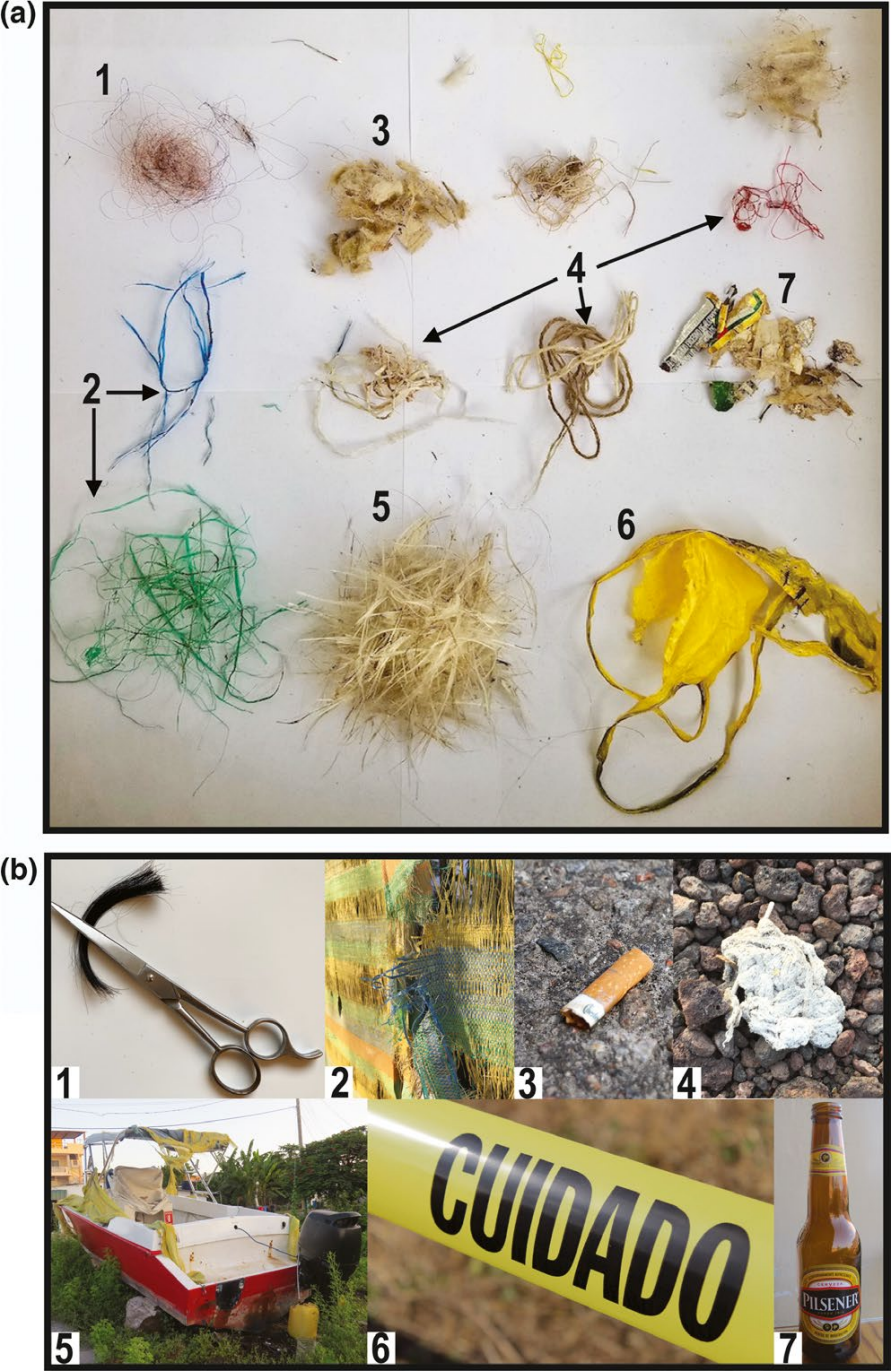
(a) Identification of anthropogenic nest materials dissected from a single *G. fuliginosa* urban nest which were sorted by material type: 1. human hair from outside salons, 2. shredded plastic tarp strands, 3. cellulose fibers from cigarette butts, 4. fibers/thread, 5. fiberglass from grounded/broken fishing boats, 6. caution tape, 7. paper shreds from bottle label. (b) Urban “sources” of anthropogenic material identified from above nest (numbered items same as above, but in urban source form)

Anthropogenic debris was not found in nonurban areas, and all nests in the urban area contained anthropogenic debris (Table 1, Figure 4). The percent of anthropogenic debris out of total nest mass varied from 3.1% to 22.7% (Supporting Information). The number of nestlings fledged declined with increasing proportion of anthropogenic material comprising total nest mass (*iii*, GLM, *χ^2^ = *13.80, *df = *1, *p* = .0002).

The most common materials found across nests were synthetic strings and fibers and synthetic stuffing followed by plastic (Figure 4, Supporting Information). In the urban area, we found and documented four cases of nest entanglement‐associated mortalities across four different nests: 1) one nestling was found hanging from ingested hair that was woven into the nest, with its leg also tangled in free hanging plastic string (Figure 3a); 2) one nestling was hanging from ingested synthetic string (Figure 3a); 3) one nestling (near fledging) was found with its leg entangled in plastic string; and 4) one adult female was found hanging, strangulated, from human hair during active nest building (Figure 3c).

## 4 DISCUSSION

Our study compared nesting success of urban and nonurban finches, within a single pair of urban/nonurban sites, and examined whether urban anthropogenic debris, which was incorporated into nests, was responsible for nestling and adult mortalities. We found that urban finches had higher nesting success than nonurban finches during a La Niña year. All urban finches incorporated anthropogenic debris into their nests and some individuals suffered associated mortalities; however, this negative consequence was not enough to offset urban nestling success. Low reproductive success in dry years is an established pattern in nonurban Darwin's finches. Consistent with the majority of urban rural comparisons (Chamberlain et al., 2009), we found that the reproductive effort, (i.e., egg laying) of urban small ground finches began earlier than in their nonurban conspecifics. Reproductive effort, as well as nesting success, was significantly higher in urban finches compared to nonurban finches; the urban area resulted in higher numbers of nests with eggs (Figure 2a), nests with nestlings (Figure 2b), and fledging success (Figure 2c). All urban finches were found to incorporate anthropogenic debris (3.1% to 22.7% of total nest mass) into their nests while nonurban nests had no anthropogenic debris incorporated. Critically, mortalities due to anthropogenic nest debris entanglement were recorded across four urban nests, affecting 18% of nests with nestlings and one female in active nest building, and debris related mortality was shown to be a cost associated with nesting in urban areas. These results suggest that despite anthropogenic debris related mortalities, small ground finches derived an overall reproductive benefit from urban habitation during a dry year, with the caveat that without replication these finding may not be generalizable to other urban sites/islands without further investigation. This benefit is perhaps associated with the earlier urban initiation of breeding and the longer sustained breeding season, which may provide less competition for resources, that is, nesting sites, which are limited in urban areas.

Our study was conducted during a La Niña year (MEI.v2; Zhang et al. 2019), which may have impacted initiation and sustainment of the breeding season in the urban area, as seen in previous studies (Boag & Grant, 1981, 1984; Koop, LeBohec and Clayton, 2013). Rainfall and the associated increase in primary productivity is known to initiate and sustain finch breeding (Boag & Grant, 1984; Gibbs & Grant, 1987; MEL.v2, 2017). Several days of heavy rain occurred the last few days of February, which seem to have been sufficient to trigger breeding, but perhaps not enough natural food resources were available to sustain breeding efforts. This short period of precipitation was preceded and followed by dry periods of low to no precipitation (Pers. obs. JAH). Existing weather station data for the island airport was only recorded for four days of the study period and was therefore not included. During the 2018 breeding season, we recorded a high percent of nest abandonments in the nonurban area: 33% of finch nests (four of 12 nests) containing eggs were abandoned. The lower limit of rainfall required to sustain finch breeding is unknown and may be impacted by rainfall the previous year or associated to carryover effects on vegetation and invertebrate communities. The previous year, 2017, was a milder La Niña year (Zhang et al., 2019), providing some indication that low primary productivity carryover was possibly affecting resource availability in the study year. However, multiyear studies are needed to determine resource effects across La Niña and El Niño years and the impact on urban and nonurban finches.

We found differences in overall reproductive output (i.e., egg laying, hatching, and nestling fledging) of urban and nonurban small ground finches in our study, which may be related to the higher food resource availability in the urban area (Lochmiller & Deerenberg, 2000) and low primary and secondary production associated with low precipitation typical of a La Niña year in the nonurban area. Urban food resources are independent of climatic variability, whereas natural food resources (e.g., seeds and insects) are lower in dry years (i.e., low precipitation, drought). Accordingly, Galápagos land nesting birds have shown reduced breeding success in dry years (Grant & Grant, 1993; Koop, LeBohec, & Clayton, 2013; McNew et al., 2019). Nonurban finch fitness, in terms of both breeding success and adult survival, is dictated by precipitation patterns and the resulting food availability (Gibbs & Grant, 1987; Koop, LeBohec, & Clayton, 2013; Grant & Grant, 2014). Therefore, future studies should examine whether the urban breeding success exceeds nonurban success in wet years and across years in order to understand the long‐term demographic patterns of urban finches. Urban and nonurban birds may face distinct and, at least, partially nonoverlapping stressors. Nonurban birds may be more impacted by climatic variables and resulting food availability, whereas urban birds may be impacted by anthropogenic stressors, such as anthropogenic debris.

We did not examine diet of urban and nonurban finches; however, previous studies have found that while human‐based food availability in urban environments can benefit adult birds, nestlings require a higher protein diet (Boag, 1987). Natural high‐protein food sources, such as arthropods, have been shown to decline with increasing urbanization (Shochat et al., 2004). Lower quality of food in urban environments has been seen to negatively affect nestling and juvenile growth in other urban bird species (Pierotti & Annett, 2001; Seress et al., 2012, 2020). Even if urban food quality is lower nutritionally than natural food sources, the quantity and consistent availability of the resources may provide short‐term benefits for survival of urban finch nestlings. However, poor nestling diet can directly impact morphological metrics later in life (Boag, 1987). While previous studies have examined the diet of finches in urban versus nonurban areas (De León et al., 2018), an examination is still needed of long‐term effects of low quality diet on urban finch demography, morphology, and overall fitness.

We found that 100% of urban nests contained anthropogenic debris, whereas no anthropogenic debris was recovered from nonurban nests. These results were likely due to local environmental variables and available resources. Abundant anthropogenic debris was available in the urban area and only two pieces of debris were seen at the nonurban area during the study period. Conversely, grasses which comprise a large proportion of nonurban nests are limited in the urban area. Consequently, 24% of urban nests were associated with debris‐related mortalities and the proportion of anthropogenic debris comprising the total nest mass impacted nestling survival. Debris‐related mortalities documented in the study were all due to ingestion/entanglement with anthropogenic debris that was found incorporated into the nest. Other studies have observed mortalities due to both entanglement and ingestion of anthropogenic debris in nestling and adult land birds (Henry et al., 2011; Mee et al., 2007; Theodosopoulos & Gotanda, 2019). However, few studies have examined the effect of debris on reproductive or nesting success in land birds (Jagiello et al., 2018), including passerines and near passerines (Antczak et al., 2010; Hanmer et al., 2017; Suárez‐Rodríguez & Macías Garcia, 2014; Townsend & Barker, 2014) as recently reviewed by Jagiello et al., (2019). Here, several types of common anthropogenic debris (e.g., plastic string, human hair, and string fibers) are frequently used by urban finches in nest building and pose a higher risk, as seen in the documented entanglement mortalities. The incorporation of anthropogenic debris into nests presents a clear cost to birds nesting in urban habitats; however, despite suffering mortalities due to entanglement, urban nestlings demonstrated higher survival than nonurban nestlings. Mortality due to entanglement/ingestion of debris in passerines is likely much more difficult to detect than in seabirds due to size of the birds and location of mortalities (i.e., tree nests vs. beached). The dearth of studies examining anthropogenic debris and associated mortalities on urban adapted passerines remains the largest hindrance to understanding the effects of anthropogenic debris on reproductive and nesting success.

Our study was conducted in an urban area with intermediate population size relative to the other human‐inhabited Galápagos Islands such as Santa Cruz and Isabel. We do not know if a higher degree of urbanization, such as in Puerto Arroyo on Santa Cruz, would yield the same beneficial result for finches. A number of species succeed and are abundant at intermediate levels of urbanization and disturbance (Ausprey & Rodewald, 2011; Perrier et al., 2018; Stracey & Robinson, 2012). However, species could reach thresholds at which they no longer benefit from increased urbanization because of further changes in habitat structure which result in a loss of resources (water, perches, nesting sites) (Blair, 1996; Lee et al., 2004). A threshold of urbanization that is detrimental for Darwin's finches might not have been reached yet. Our study was conducted in a single year without replication across sites due to lack of additional urban cities on San Cristóbal. The only other concentrated human population is El Progreso, a rural farmland village with a population size of 535, and consequently, replication for this study is not possible within the same island (INEC, 2015). Duplication of this study on a different Galápagos Island with urbanization, such as Puerto Ayora on Santa Cruz and Puerto Villamil on Isabela, could uncover other factors impacting urban finch success. In addition to direct anthropogenic threats, Darwin's finches in both urban and nonurban areas are also affected by an introduced nest parasite, *Philornis downsi*, which can affect their reproductive success (Kleindorfer & Dudaniec, 2016; Knutie et al., 2016; McNew & Clayton, 2018). We did not consider possible effects of *P. downsi* in our study because we lacked the power to detect an effect of the parasite (due to the low nest success in the nonurban area). Future studies should examine whether the costs and benefits of urbanization for the finches vary across different Galápagos Islands as well as study the interactions between urbanization and *P. downsi* in limiting reproductive success of Darwin's finches.

## CONFLICT OF INTEREST

The authors declare that they have no conflict of interest.

## AUTHOR CONTRIBUTIONS

**Johanna A. Harvey:** Conceptualization (equal); Data curation (lead); Formal analysis (lead); Investigation (lead); Methodology (equal); Project administration (equal); Supervision (equal); Validation (equal); Visualization (lead); Writing‐original draft (lead); Writing‐review & editing (lead). **Kiley Chernicky:** Investigation (supporting); Project administration (supporting); Supervision (supporting); Writing‐review & editing (equal). **Shelby R Simons:** Investigation (equal); Writing‐review & editing (equal). **Taylor B Verrett:** Investigation (equal); Writing‐review & editing (equal). **Jaime A Chaves:** Project administration (equal); Resources (equal); Writing‐original draft (supporting); Writing‐review & editing (equal). **Sarah A. Knutie:** Conceptualization (lead); Data curation (equal); Formal analysis (equal); Funding acquisition (lead); Investigation (equal); Methodology (equal); Project administration (lead); Resources (lead); Supervision (equal); Visualization (equal); Writing‐original draft (equal); Writing‐review & editing (equal).

## 5 ETHICS STATEMENT

All applicable international, national, and/or institutional guidelines for the care and use of animals were followed. All bird handling and work was conducted according to approved University of Connecticut IACUC (Institutional Animal Care and Use Committee) protocols (No. A17‐044). Our work was done under GNP permits PC 03‐18 and Genetic Access permit MAE‐DNB‐CM‐2016‐0041.

## Supporting information

Supplementary Material

## Data Availability

All raw data are available on FigShare (https://doi.org/10.6084/m9.figshare.12928838.v2).
