# Supplementary material for: Urban living influences the nesting success of Darwin’s finches in the Galápagos Islands
Source: Ecol Evol. 2021 Mar 16;11(10):5038–48. doi: 10.1002/ece3.7360 (PMC8131787; doi:10.1002/ece3.7360)
Supplement: Supplementary file 1 — Supplementary Material [file ECE3-11-5038-s001.docx]

**Supporting Information.** Qualitative data for materials dissected from *G. fuliginosa* urban nests containing anthropogenic materials, where anthropogenic materials are identified and noted with a 1 for presence and 0 for absence.

| Nest  ID | Total nest mass (g) | Anthro-pogenic material % | Anthropogenic material type | | | | | | | |
| --- | --- | --- | --- | --- | --- | --- | --- | --- | --- | --- |
|  |  |  | Metal | Fiberglass | Synthetic stuffing | String fibers | Paper | Cig-arette | Human hair | Plastic |
| 01 | 16 | 18.8 | 0 | 1 | 1 | 1 | 0 | 0 | 1 | 1 |
| 02 | 30 | 13.3 | 0 | 1 | 0 | 1 | 0 | 0 | 1 | 1 |
| 03 | 49 | 4.1 | 0 | 1 | 1 | 1 | 0 | 0 | 0 | 0 |
| 04 | 24 | 12.5 | 0 | 0 | 1 | 0 | 0 | 0 | 1 | 0 |
| 05 | 22 | 22.7 | 0 | 1 | 1 | 0 | 0 | 0 | 1 | 1 |
| 06 | 7 | 14.3 | 0 | 1 | 1 | 1 | 0 | 0 | 1 | 0 |
| 07 | 54 | 11.1 | 1 | 1 | 1 | 1 | 1 | 1 | 0 | 1 |
| 08 | 27 | 3.7 | 0 | 1 | 1 | 1 | 0 | 0 | 0 | 0 |
| 09 | 21 | 19.0 | 0 | 0 | 1 | 1 | 0 | 1 | 1 | 1 |
| 10 | 33 | 9.1 | 0 | 0 | 1 | 1 | 0 | 1 | 0 | 1 |
| 11 | 43 | 9.3 | 0 | 1 | 1 | 1 | 0 | 0 | 1 | 1 |
| 13 | 32 | 3.1 | 0 | 0 | 1 | 1 | 0 | 1 | 1 | 0 |
| 14 | 35 | 11.4 | 0 | 1 | 1 | 1 | 0 | 1 | 1 | 1 |
| 16 | 18 | 5.6 | 0 | 0 | 1 | 1 | 0 | 1 | 1 | 0 |
| 17 | 28 | 7.1 | 0 | 1 | 1 | 1 | 0 | 0 | 0 | 0 |
| 18 | 43 | 18.6 | 0 | 1 | 1 | 1 | 0 | 1 | 0 | 1 |
| 19 | 28 | 3.6 | 0 | 0 | 1 | 0 | 0 | 0 | 1 | 1 |
| 20 | 31 | 6.5 | 0 | 0 | 0 | 1 | 1 | 0 | 0 | 1 |
| 21 | 33 | 9.1 | 0 | 0 | 0 | 1 | 0 | 1 | 1 | 1 |
| 22 | 33 | 6.1 | 0 | 0 | 1 | 1 | 0 | 0 | 0 | 1 |
| 23 | 40 | 10.0 | 0 | 1 | 1 | 1 | 1 | 1 | 1 | 1 |
